# Cases of Poisoning by Arsenic—Treated by the Hydrated Peroxide of Iron

**Published:** 1840-10-24

**Authors:** T. T. Smiley, J. M. Wallace


*Cases of Poisoning by Arsenic—Treated by the Hydrated Peroxide of Iron.* By T. T. Smiley, M. D., and J. M. Wallace, M. D.

On the 3d day of January, 1840, the family of Mr. Constant Gigon, residing at No. 148 Pine street, Philadelphia, was poisoned by eating a pudding made of Indian meal, in which arsenic had been mixed, probably for the purpose of destroying rats. The precise quantity of arsenic contained in the pudding could not be ascertained; but, judging by the effects produced, must have been very great. The whole family, consisting of eight persons, was affected with symptoms more or less violent. An hour after the pudding had been eaten, on finding a general sickness pervading the family, a dose of ipecacuanha had been administered to each of the individuals affected, by Mr. Zollickoffer, an apothecary residing in the neighbourhood.

On the arrival of Dr. Smiley, who was first called in, poisoning from arsenic was at once suspected,—and on that supposition copious drafts of warm water, with sweet oil, were administered, and followed by whites of eggs in large quantities.

During the operation of the foregoing remedies, given with a view to evacuate the stomach, and remove the poison as thoroughly as possible, Mr. Zollickoffer was requested to prepare a large quantity of the hydrated peroxide of iron, for the purpose of being administered as an antidote to that portion of arsenic which always in such cases remains in the stomach, and cannot be entirely evacuated by vomiting.

As soon as the hydrated peroxide of iron could be prepared, it was administered in tablespoonful doses, at short intervals, to all the patients, and as often as rejected by vomiting, was immediately repeated. In nearly all the patients an almost entire subsidence of the most violent symptoms soon followed, and the burning sensation of the œsophagus and stomach greatly diminished. The result of the subsequent treatment will appear by the following detail of each particular case.

1. Constant Gigon, aged thirty years, slender frame, and delicate constitution. Symptoms—extremities cold; pulse at the wrist scarcely perceptible; extreme anxiety of countenance; constant vomiting, with great thirst, and violent pain in the epigastrium, increased by pressure; spasms in the lower extremities; no priapism or purging; intellect perfectly clear; tongue covered with a thick white fur, through which the red papillæ stood prominently, very much resembling the appearance of the tongue in severe cases of scarlatina. The hydrated peroxide of iron could not be retained by the stomach more than a few minutes. Ammon. carb. in mucil., with brandy, appeared to check the vomiting; but when the hydrated peroxide of iron was again given, it was immediately rejected. Brandy enemata were ordered every hour. Sinapisms over the stomach, and frictions, with capsicum and brandy; warm bricks kept constantly to the feet. The heat acted only as it would have done on a dead body. No reaction took place, and the patient died about seven hours after eating the poisoned pudding. His intellect was clear to the last moment, and for a short time before his death he complained only of feeling cold. No evacuation from the bowels at any time.

*Autopsy sixteen hours after death.*—Present, Drs. Peace, Pepper, and Kirkbride. All the muscles were very firmly contracted, though the body had been kept in a warm room. There was not the slightest relaxation of the walls of the abdomen, which were drawn inward, and so elastic as to feel stretched like the head of a drum. The stomach and duodenum were first removed, with a ligature applied to each orifice to retain the contents. On being opened the stomach was found to contain nearly a pint of a brown viscid fluid, which was reserved for chemical examination. The whole mucous membrane was injected, and of a bright scarlet colour, with effusion of lymph in some parts. No ulceration. On attempting to detach strips, the stomach was found thickened and *softened* for the space of three inches round the pylorus. The mucous follicles enlarged. Several bright patches, from an inch to two inches in diameter, formed round a central point of irritation. Around the pylorus also were several ecchymosed spots, from half an inch to an inch in diameter, and a few white points on the mucous membrane. The duodenum was injected only for an inch or two beyond the pylorus, but in a much less degree than the stomach. The mucous membrane of the fauces and œsophagus was considerably injected. The other intestines in a perfectly healthy state.

2. Madeline Cetter, aged twenty-four years, symptoms at first less severe than the above. Afterwards incessant vomiting came on. The hydrated peroxide of iron could not be retained on the stomach, and the slightest portion of any fluid excited vomiting. About six hours after being poisoned, her intellect became torpid, so that she could with difficulty be roused. When disturbed was exceedingly restless, and complained of constant burning in the epigastrium. Brandy enemata, frictions, warmth, &c., failed to produce reaction, and the patient died nine hours after being poisoned.

*Autopsy thirteen hours after death.*—The same powerful contraction of the muscular system as in the foregoing case. The stomach contained half a pint of yellowish, turbid fluid. The mucous membrane much less injected than in case first, though near the pylorus there was a spot two inches square, intensely scarlet, and very minutely injected. Several smaller spots, similar in appearance, and from two to three lines in extent, were found in other places. The large veins could be seen through the membrane very much distended. The duodenum, and other intestines, not in the least affected.

3. Marie M. Carter, aged fifty-four years, very feeble; tongue did not show the red papillæ to the same extent as the others, but was heavily coated with white fur; vomited freely at first, but soon retained the hydrated peroxide of iron. No subsequent gastritis. Perfectly recovered in a few days.

4. Mrs. Gigon, aged twenty-five years. Symptoms at first not very severe, but succeeded by great prostration, attended with shivering; vomiting not very frequent, and consequently the iron was pretty well retained. On the second day great irritability of the stomach, which was allayed by laudanum and ice water. Pulse inflated; tongue thick; pain in epigastrium; no purging; warmth to the extremities; sago diluents, &c. Sixth day, broth. Seventh day, chicken, &c. Perfectly recovered.

5. Gustavus Gigon, aged twenty-one years. Symptoms generally not severe, but complained of great burning in the stomach, which was immediately relieved by the hydrated peroxide of iron. The remedy was freely administered, and remained on the stomach. Said it stopped the burning, and asked for more. Pulse quick, full, and soft. Complained for several days of uneasiness in the epigastric region. No purging. Tongue heavily coated with white fur, which did not clear off before the eighth day. Appetite good, however. Soon restored to perfect health.

6. Child aged twenty-eight months. Severe vomiting; coldness of the skin and extremities; jaws firmly clenched, and with difficulty opened to administer the hydrated peroxide of iron. Insensibility. Countenance deathlike. Vomiting still continued, but the antidote was repeated until it remained on the stomach. Thirst excessive. Second day: tongue furred, and marked with red papillæ more than the others. Some tenderness of the epigastrium on pressure. Tendency to coma with constant thirst. Ice water, warmth to extremities, &c. Third day: vomiting; allayed by a blister on the epigastrium, and lime-water and milk internally. Rapid convalescence and entire recovery.

7. Child aged sixteen months, not weaned. Symptoms at first nearly similar to the preceding case. The hydrated peroxide of iron was retained on the stomach, and recovery soon took place.

8. Margaret Maxwell, cook. Ate of the pudding after the rest of the family. Suddenly seized with chilliness and nausea. The hydrated peroxide of iron retained on the stomach. The tongue coated like the others. Pain in the head. Inflated pulse. Got up next morning, but became giddy, and went again to bed. No striking symptoms. In a few days was perfectly well.

*Remarks.*—In the two cases which proved fatal, death appears to have ensued from the direct effects of the poison on the nervous system. It might even be doubted whether the injury sustained by the stomach in either case was sufficient to have produced death by subsequent inflammatory action, and possibly both these patients might have recovered, provided the direct shock to the system could have been obviated, and reaction produced.

It is worthy of observation that every one of the patients who were able to retain the hydrated peroxide of iron on the stomach speedily recovered, and were restored to perfect health, with the entire exemption from any symptom indicating the existence of chronic inflammation of the stomach which has heretofore so uniformly followed poisoning from arsenic in the few cases which have not proved immediately fatal.

The great and almost immediate alleviation of the urgent symptoms which followed in every instance in which the hydrated peroxide of iron could be retained on the stomach, affords the strongest evidence that the beneficial results ascribed to that remedy in such cases have not been overrated. Fortunately, few instances have occurred in which the virtue of that antidote could be tested on so large a scale.

*Note.*—The contents of the stomachs of the two patients who died were submitted to a careful chemical examination by Dr. Rogers, and the existence of arsenic in them placed beyond a doubt. A large quantity of metallic arsenic was procured both from the contents of the stomachs and the remains of the pudding.

*Philadelphia*, Nov., 1840.

### PHILADELPHIA DISPENSARY.

*Report of Cases treated in the North Middle District during the months of August and September.*

| | | |
|---|---|---|
| | Whole number | 73 |
| Of these, | Recovered | 67 |
| | Died | 3 |
| | Relieved | 3 |
| | Total | 73 |

In the report of this district for July, it appeared that the prevailing disease in the northern and middle portions of the city, was an affection of the bowels. The tendency to this was strongly marked during the whole of the month of August, when it disappeared, and fever of an intermittent character assumed its place. The paroxysms of this were generally slight, and readily yielding to the influence of quinine, given in large and frequent doses. Towards the latter part of September, few cases of intermittent remained, and at the present time it has entirely disappeared from those parts of the district attached to the Dispensary. C. N. Berkeley.

